# “She mimicked the manipulations on my hand”: fostering embodied care among children with recurrent acute respiratory tract infections in Southern China

**DOI:** 10.1186/s12906-024-04660-6

**Published:** 2024-10-07

**Authors:** Lingjia Yin, Bei Chang, Cecilia Stålsby Lundborg, Darong Wu, Helle Mølsted Alvesson

**Affiliations:** 1 Department of Global Public Health, Karolinska Institutet, Stockholm, Sweden; 2 State Key Laboratory of Dampness Syndrome of Chinese Medicine, The Second Affiliated Hospital of Guangzhou University of Chinese Medicine, Guangzhou, China; 3 Outcome assessment research team in Chinese medicine, Guangdong Provincial Hospital of Chinese Medicine, Guangzhou, China; 4 The Second Clinical Medical College, Guangzhou University of Chinese Medicine, Guangzhou, China; 5 Guangdong Provincial Key Laboratory of Clinical Research on Traditional Chinese Medicine Syndrome, The Second Affiliated Hospital of Guangzhou, University of Chinese Medicine, Guangzhou, China

**Keywords:** Pediatric *Tuina*, Complementary and alternative medicine, Embodiment, Caregivers’ experience, Recurrent respiratory tract infections

## Abstract

**Introduction:**

When young children experience recurrent respiratory infections, caregivers face the challenge of preventing new episodes whilst maintaining close rapport with their children. Pediatric massage, such as pediatric *Tuina*, entails soft massage of the skin, administered by trained providers. This non-pharmaceutical measure is used to prevent new respiratory infections in China. The aim of this study is to deepen our understanding of caregivers’ experiences and perceptions of providing pediatric *Tuina* treatment to their children with recurrent respiratory tract infections.

**Methods:**

A qualitative study, based on semi-structured interviews, was conducted in accordance with the Consolidated Criteria for Reporting Qualitative Research checklist. Sixteen mothers from Southern China, whose children had received pediatric *Tuina* for recurrent respiratory tract infections, participated online. Analysis was conducted according to the principles of reflexive thematic analysis, using the NVivo qualitative research software.

**Results:**

The overarching theme was “Fostering embodied care with children”. Caregivers assessed pediatric *Tuina* by hearing others’ experiences of pediatric *Tuina*, as well as observing the manipulations on their child’s body and their bodily reactions during pediatric *Tuina* sessions. Caregivers also closely observed children’s bodily changes after pediatric *Tuina* sessions. Embodied attachment between children and adults was nurtured through the pediatric *Tuina*. Compared to other treatments or medical consultations, children were more relaxed and more involved in embodied care, which involved direct skin touching and verbal communication from the pediatric *Tuina* provider. Children also took the initiative to bring pediatric *Tuina* into their family life, by asking caregivers to perform it on them and mimicking the manipulations on the caregivers’ hand.

**Conclusions:**

Pediatric *Tuina* served as a means of interaction between children and adults, fostering an embodied care on both a physical and emotional level. Beyond its potentially preventive effect on recurrent respiratory tract infections, pediatric *Tuina* could be a support for parents of children with recurrent or chronic disease at home.

**Supplementary Information:**

The online version contains supplementary material available at 10.1186/s12906-024-04660-6.

## Introduction

Recurrent respiratory tract infections (RRTIs) are common in young children worldwide and are among the leading causes of hospital visits and school absence [1, 2]. Although RRTIs mainly involve the upper respiratory tract and are associated with fever, cough, running nose and sore throat, there is no universal consensus on its definition. However, it is commonly accepted that recurrence describes more than six episodes in a year [3]. In China, the estimated annual prevalence of RRTIs among kindergarten children was around 25% in 2015 and 2019 [4, 5]. Of all children who visited the outpatient department in a tertiary hospital in Beijing, around 60% had a respiratory tract infection, with more than 50% coming for a repeat visit [6]. RRTIs represent a significant burden on the children, their families and the healthcare services. They involve multiple hospital visits/admissions, healthcare costs, transportation costs, work/school absenteeism and overuse of antibiotics [2, 7, 8]. Parents with a child who is suffering from RRTIs have a higher risk of depression and anxiety and lower quality of life [9].

Antibiotics are often used to treat respiratory tract infections, although most infections are self-limiting. It has been reported that antibiotics have been used too often among children with RRTIs [7]. The overuse of antibiotics is associated with antimicrobial resistance, ineffectiveness of treatment and the increased risk of adverse effects [10]. Increased antimicrobial resistance is the cause of severe infections, complications, extended hospital days and increased mortality [10]. Therefore, it is important to prevent the recurrence of respiratory tract infections and adopt complementary treatment, not only in treatment but also in prevention. The conventional means of prevention include quitting smoking, getting vaccinated, avoiding allergens and practicing good hygiene [7]. In some cases, pidotimod, a synthetic molecule, which may stimulate the immune system, is recommended to prevent RRTIs [3]. However, it is not recommended for routine use [3] and the existing evidence only supports its administration to children from the age of 3 upwards [11]. Clinically, traditional, complementary and integrative healthcare (TCIH) has been employed by some practitioners to prevent new episodes of RRTI in children, to overcome the shortcomings of both antibiotics and pidotimod. However, the available evidence does not support this use [3]. Homoeopathy, natural substances and phytotherapy are the common TCIH practices for RRTI prevention in children in Western countries, whereas Chinese medicine practitioners often choose pediatric Tuina.

**Fig. 1:**
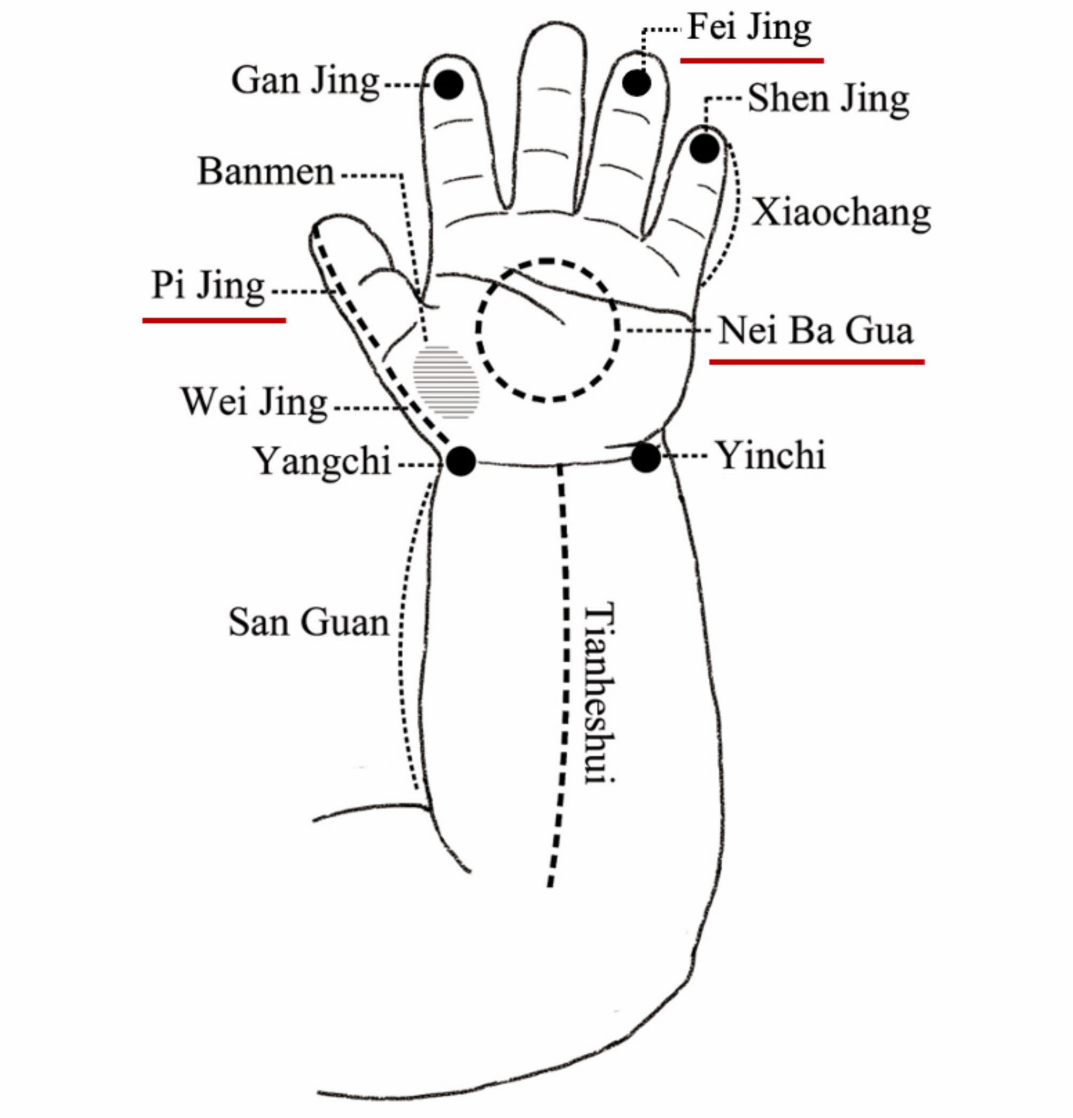
Three most common acupoints on hand in treating children’s RRTIs

While touch therapy and baby massage are well known in many parts of the world, pediatric *Tuina* (PT) is less known outside China. In Chinese medicine, *Tuina* is not only used to relax the patient or treat diseases of the musculoskeletal system and connective tissue, but also to re-balance the energy flow of various systems, such as the digestive and respiratory tracts, by stimulating the acupoints [12]. Therefore, in China PT has also been used to treat some internal disorders in children, such as cough and diarrhea [13]. In PT, the manipulations are different from other types of pediatric massage. Acupoints are mainly chosen from the left hand and forearm, according to the TCM patterns of the child, and are stimulated with light and stable strength, but fast speed. The most common acupoints used to treat children’s RRTIs are Ji (spine), Zu San Li (ST 36), Fei Jing (on the ring finger), Pi Jing (on the thumb), Nei Ba Gua (on the palm) and Fei Shu (BL 13) [14] (Fig. 1). Whilst English language journal articles on PT are scarce, numerous studies have been published in Chinese, suggesting the positive effects of PT on children with RRTIs [15–19]. However, two systematic reviews of these original studies indicated that the evidence does not support a recommendation to use PT in the prevention of RRTIs [20, 21]. This is mainly due to the small sample sizes and methodological shortcomings. Studies with large sample sizes and high methodological quality are therefore required to further explore the effect of PT on RRTI prevention. In our previous study, which included more than 2,000 children with RRTIs, we found that having six or more pediatric Tuina sessions within one year is associated with a decrease in RRTIs in children in the following two years [18]. Although it is emphasized, in evidence-based medicine, that patient values and preferences are one of the key components [22], little is known regarding caregivers’ perceived experience of PT in preventing children’s RRTIs. Thus, in this study, our primary aim is to understand the experiences of PT from the caregivers’ perspective.

The theory of embodiment may help us deepen our understanding of caregivers’ experience and perceptions of PT. Embodiment is a dynamic conceptual theory, developed in the 20th century by Maurice Merleau-Pongty to oppose Cartesian dualism, where mental phenomena are considered non-physical, or the mind and body are not identical [23]. Its premise is that human experiences or emotions are situated throughout the body [24]. There is a paucity of literature describing the utilization of embodiment theory to explore children’s illness [25, 26]. In contrast, there is more literature utilizing embodiment theory to explore patients’ experience of manual therapy, such as massage and touching [27–29]. This may be due to the body-centered characteristics of manual therapy. In China, there is no belief in the Cartesian body [30], but rather in the concept of the unity of body and mind, or even the unity of body and environment [31]. Although the theory of embodiment was generated in western countries, it’s meaning is the same as Chinese medicine’s conceptualization of the unity of body and mind. While the concept of unity of body and mind is a cornerstone of Chinese medicine [32], it is still under-represented in Chinese medicine research. Therefore, our aim of this study is to deepen the understanding of caregivers’ experiences and perceptions during and after the use of PT on their children with RRTIs, through the lens of embodiment.

## Methods

This study has been conducted and reported in accordance with the Consolidated Criteria for Reporting Qualitative Research (COREQ) checklist [33].

### Study setting

The study was conducted in Dongguan, Guangdong Province, Southern China. It is one of the world’s largest manufacturing bases and is known as the “world’s factory” in China. It maintains an annual net population inflow of more than 100,000 people, which has increased the household population by 690,000 in five years [34]. According to the demographic data, in 2021 Dongguan was the third largest city in Guangdong Province, with a population of over 10 million [35]. It is a very young city with a large workforce with a low education level [36]. In China, Chinese medicine and biomedicine are both integrated in the healthcare system. People may seek Chinese medicine in Chinese medicine hospitals, in the Chinese medicine department of general hospitals and at community healthcare centers. There are 66 general hospitals, 8 Chinese medicine hospitals and 395 community healthcare centers in Dongguan [37]. Around 3000 healthcare workers are employed in the Chinese medicine hospitals [37]. This study was conducted in the Department of Pediatrics in Dongguan Kanghua Hospital, which is a private general hospital. Three nurses from this department have been trained in the practice of PT and provide PT therapy daily. Children receive PT in a clean and bright room with chairs where they can sit and a bed where they can lie down. The caregivers are always present during the PT sessions.

### Study design and participant selection

Participants of this study were recruited among mothers of children enrolled in a clinical trial, which aimed to compare gut microbiota patterns before and after PT therapy [38]. Face to face interviews were initially planned, but because of the COVID-19 pandemic, digital interviews through WeChat voice call were carried out instead. A convenience sampling strategy was used because of COVID-19 restrictions. In the clinical trial, each child received PT from a trained PT practitioner, belonging to the research group, three times per week for one month during November 2020 to January 2021. A total of 20 children were included in the trial, based on the following inclusion criteria: They were (i) diagnosed with acute RRTIs, (ii) aged 36–72 months, (iii) ate properly and defecated regularly, and (iv) had caregivers who could cooperate in the collection of feces samples. In addition, none of the children had allergic disease, such as asthma or eczema, or other diseases which could cause disorders of the gut microbiota, such as diarrhea. The first author (LY) contacted these 20 mothers by phone. Of those contacted, sixteen agreed to participate in the study (Table 1). Four did not agree due to lack of interest or time. The participants chose the interview time at their convenience. The interviews were conducted between January and June in 2021.

**Table 1:** Characteristics of participants and their children who received the one-month pediatric *Tuina*

ID	Mothers’ characteristics			Children’s characteristics		Length of Interview (minutes)
	Housewife	Heard of PT before	Number of children	Used PT before	Main recurrent respiratory problems reported	
1	Yes	Yes	1	Yes	Coughing and wheezing	55
2	Yes	Yes	2	No	Bronchitis	56
3	No	Yes	1	Yes	Coughing, rhinitis	47
4	No	Yes	2	No	Asthmatic bronchitis	53
5	No	Yes	1	No	Coughing and wheezing	51
6	No	Yes	2	No	Common cold, fever	46
7	No	Yes	2	Yes	Acute pharyngitis	48
8	No	Yes	1	No	Acute tonsillitis	50
9	Yes	Yes	2	Yes	Coughing	62
10	Yes	Yes	2	Yes	Rhinitis	35
11	No	Yes	1	Yes	Acute tonsillitis	45
12	No	Yes	2	No	Coughing, throat pain	41
13	No	Yes	1	No	Coughing, acute tonsillitis	60
14	Yes	Yes	2	Yes	Coughing, bronchitis	43
15	No	Yes	2	Yes	Asthmatic bronchitis	50
16	No	Yes	1	No	Common cold, pneumonia	47

### Data collection

An interview guide was developed based on the literature on RRTIs and included six topics: The disease story of the child; the caregivers’ healthcare seeking experiences, their views on PT, their experiences with PT, the results after PT and the reasons for participating in the trial. For each topic, several open-ended and probing questions were asked. Two pilot interviews were conducted to test the feasibility of the guide. Since only minor revisions were made for comprehension, these two pilot interviews are included in the analysis. LY conducted all of the interviews according to the interview guide. Although LY is a novice in the qualitative research field, she had prior experiences of qualitative research methods and of conducting interviews. In order to secure the quality of data collection, the first five interviews were transcribed in Chinese and translated into English before the remaining interviews were conducted. Based on the English transcripts, researchers had discussions about the interview technique, such as how to ask a better probing questions. Each interview lasted between 35 and 60 min, as some mothers are extremely talkative while some mothers are the opposite. The average length of interview was 49 min, while only one mother had the interview less than 40 min and two mother spent more than one hour in the interview. Interviews were audio recorded by a Samsung voice recorder manufactured by Huizhou Samsung Electronics Co., Ltd. and complemented by researcher notes written during and after the interviews.

### Data analysis

The interviews were transcribed verbatim in Chinese by LY and checked by BC. Analysis was conducted according to the principles of reflexive thematic analysis [39, 40], through which we explored the data inductively by means of utilizing the researcher’s subjectivity as the primary tool. Reading and re-reading of the transcripts was carried out in the initial period of analysis. After gaining familiarity with each of the interviews, coding was conducted line-by-line by LY via the NVivo qualitative research software (QSR International Propriety Limited, 2019). To maintain the contextual meaning [41, 42], the coding was based on the Chinese transcripts, but English code names were used to facilitate discussions among researchers. The codes were discussed to identify the semantic and latent patterns of the caregivers’ experiences with the use of PT on their children. At this point, it was found that both bodily and emotional patterns were correlated with the experience of PT. Therefore, the decision was made to apply the theory of embodiment. The data was then revisited and the transcripts recoded through the lens of embodiment. Initial themes were formed by LY in consultation with HMA. The themes were then reviewed and refined through further discussions among the researchers. Finally two sub-themes were developed under the framework of embodiment [24].

## Results

The overarching theme of “Fostering embodied care with children” is constructed from two sub-themes: (1) Assessing PT through embodied observations and (2) Nurturing embodied attachment with children. (Table 2.)

**Table 2:** Overarching theme, sub-themes and categories

Overarching theme	Fostering embodied care with children	
Sub-theme one	Assessing PT through embodied observations	• Trusting others’ embodied treatment experiences
		• Observing soft and gentle manipulations on the body
		• Monitoring children’s bodily reactions during the PT sessions
		• Parental expected and unexpected bodily changes after PT
Sub-theme two	Nurturing embodied attachment with children	• Observing rapport between children and PT providers
		• Having interest in doing PT for children at home
		• Noticing children took the initiative in involving PT in family life

### Sub-theme 1. Assessing PT through embodied observations

The safety of their children was an important concern for caregivers during their healthcare decision-making process. For the interviewed mothers, the safety of PT was expected to be high. No specific worries about using PT for their children were emphasized. Mothers assessed PT before, during and after the one-month PT sessions, through several approaches.

In most cases, mothers described hearing about PT from trusted friends and relatives, with whom they would exchange children’s illness stories and embodied treatment experiences. When positive treatment results were shared, the caregivers were likely to try the suggested treatment.

*“A friend in our community recommended it. She said she took her child to do Tuina. Her child also suffered from adenoidal hypertrophy. After the Tuina therapy*,* she had fewer common colds.”* (ID: 15, previous PT).

Mothers observed the PT manipulations of their children’s bodies carefully. They found that the manipulations were soft and gentle, and were mainly performed on their children’s hands and backs. Mothers appreciated that the therapy was conducted in a clean room, following clear information about the procedures of PT.

*“I saw he (PT provider) gently did the manipulation Tui on the forehead. At that time*,* I felt that it must be safe.”* (ID: 6, no previous PT).

In addition, mothers observed their children’s reactions during the PT sessions. Most mothers reported that their children enjoyed PT, finding it to be fun or to provide comfort. A few mothers described their children disliking PT, since they felt bored and could not sit still, disliked being touched by others, or felt itchy or sore with some manipulations. However, these mothers still hoped that their children would complete the PT sessions, and used toys or cartoon videos to distract them into cooperation.

*“I know nothing about those manipulations*,* I can only see the reaction of my child. When I took him to do PT*,* he was cooperative and happy. It seemed acceptable to me.”* (ID: 10, previous PT).

*“He kept asking ‘is it over?’ He couldn’t sit still. He was cooperative if I played videos.”* (ID: 6, no previous PT).

During and after the one-month PT sessions, mothers also monitored their children’s bodily changes. They reported expected changes, such as reduced coughing frequency, longer intervals between episodes, and quick recovery from later episodes. Unexpected changes reported included better complexion and improvement in eating, sleep or defecation (Table 3.). Some unexpected changes were observed despite the absence of any changes in coughing patterns. However, there were two mothers (ID: 04, 16) who reported no physical changes at all.

**Table 3:** Parental quotes regarding expected and unexpected bodily changes during and after PT

Changes	Sub-changes	Quotes
Expected changes	Reduced coughing frequency	*“She always had yellow phlegm*,* purulent phlegm*,* very yellow. When she had PT*,* the color would turn white gradually; the frequency of coughing would also decline. Before PT*,* she coughed day and night. After PT*,* she had less cough in the evening and only a little bit during the day.”* (ID: 9, previous PT)
	Longer intervals between episodes	*“At least*,* after that cold and cough*,* which lasted for over a month*,* up to now*,* for at least this one month*,* she has not been sick.”* (ID: 1, previous PT)
	Recovered quickly in the next episode	*“Afterwards*,* after doing the massage*,* he seldom gets sick. When he does get sick*,* he just has fever and the fever disappears on the next day. I haven’t been worried about him for a while until now.”* (ID: 11, previous PT)
Unexpected changes	Complexion	*“Do you know what the most noticeable change is every time she finishes her PT session? It’s that her complexion looks better.”* (ID: 1, previous PT)
	Food intake	*“His appetite became good after several times of PT. I told Dr. Li (PT provider, pseudonym) that I did not see some changes on his cough yet*,* but his appetite became good unexpectedly.”* (ID: 6, no previous PT)
	Sleeping	*“She slept better. Before*,* during sleeping*,* she would crawl with her eyes closed. Moving from the head of the bed to the end of the bed. During PT*,* she slept continuously until the morning without moving. She could wake up right at the place she fell asleep.”* (ID: 2, no previous PT) *“She slept much more stable during this half year. Before*,* she sweated a lot while sleeping*,* especially the first half of the night. No matter how cool it is*,* her head would sweat a lot. Now the condition became better.”* (ID: 13, no previous PT)
	Defecation	*“Before PT*,* his feces were hard. After PT it became better*,* not that hard. The color is green-yellow*,* much more normal.”* (ID: 3, previous PT)

Mothers were uncertain as to whether the bodily changes in their child could be ascribed to PT or simply to time passing and the viral infection declining. They reflected that the infection-related improvements may be due to multiple factors, such as their adjustment of caring or feeding patterns, better sleep, an increased amount of outdoor activities, natural processes related to growing older or the effect of PT.

*“After doing PT*,* he seldom became sick. If he was sick*,* having fever*,* he would recover on his own on the next day. He is like this until now. I don’t know if it is the effect of PT or because he is growing older and has a stronger immunity.”* (ID: 11, previous PT).

Among mothers who observed no changes in coughing, some believed that it was because their children had not had enough PT sessions. They believed that PT would eventually be effective, if they continued to do it.

### Sub-theme 2. Nurturing embodied attachment with children

PT sessions involve both direct skin-to-skin contact and verbal communication between children and PT providers. The practice creates an opportunity for children to form a rapport with the PT providers. Most children expressed to their mothers that they liked to be with the PT provider and asked when they would receive PT again. One mother thought that the person who provided the PT influenced her child’s willingness to receive it.

*“She liked (PT). That young man (PT provider)*,* she liked. Once*,* she had something tasty*,* she said she wanted to share it with him (PT provider). She often asked shall we go to do PT?”* (ID: 15, previous PT).

*“The person who performs pediatric massage has an influence on my son. If he likes that person*,* he will be very willing to go. He feels that he is going to play with that person. If he does not like that person*,* he will not be willing to go. So he liked Dr. Li (PT provider*,* pseudonym) very much and would like to go and play with him. Some PT practitioners in for-profit private sectors do not communicate very well with my son*,* or sometimes their techniques are not good and cause pain to him*,* thus he is unwilling to do PT.”* (ID: 14, previous PT).

Mothers also reported that children seemed to be less nervous attending PT than when seeing a doctor in usual circumstances.

*“Doing massage is quite relaxing*,* and the atmosphere is not intense at all. It doesn’t feel like going to get treated. The children feel like they’re being touched by someone else. Yeah*,* it’s neither painful nor itchy. Of course*,* there might be some discomfort in certain areas*,* but it’s not to the extent of being painful. However*,* when he enters a hospital environment*,* he still gets nervous. If it’s not in (hospital)…. in some other different context*,* it’s certainly a different experience.”* (ID: 4, no previous PT, but his older sister had).

In addition, mothers said that they had considered learning PT themselves when they saw the provider performing it on their children. They expressed an interest in performing PT on their children at home on a daily basis to prevent recurrent RRTIs. Some of them believed that PT skills can be obtained if they put in enough effort. Others said that the manipulations themselves seemed easy, but were concerned about the speed at which they were undertaken and the need for them to be conducted repeatedly for a long time.

*“(laughter) I was thinking whether I can learn how to do the manipulations and then do it for her at home.”* (ID: 5, no previous PT).

“I learned that manipulations like Qing Tian He Shui (清天河水) and Tui Liu Fu(退六腑)may help dealing with fever. I tried on my children when they had fever.” (ID: 2, no previous PT).

Several children were also said to have taken the initiative to bring PT into their family life. They required their mothers to perform PT before they went to sleep. One mother reported that her daughter learned to do PT on her own hand through mimicking the professional PT provider.

*“During that time*,* she always asked me to do the spine pinching for her when we came back home.”* (ID: 5, no previous PT).

*“Recently*,* when she had PT from Dr. Li (PT provider*,* pseudonym)*,* she asked me to show my hand and she mimicked the manipulations on my hand and said she did Tuina for me”* (ID: 1, previous PT).

## Discussion

Understanding caregivers’ experiences and perceptions of PT during and after its delivery to children with RRTIs is important, to optimize PT as a supportive measure for parents at home.

From the caregivers’ perspective, PT was seen as a safe practice for children. This is in accordance with the findings of a qualitative study in Hong Kong exploring parents’ experiences with PT in children with attention deficit hyperactivity disorder, which reported that parents considered PT to be safe, with no or minimal side effects [43]. A study in Australia also reported that parents perceived TCIH practices, including massage, to be safe [44]. They thought it was natural to the body and had no worries about side effects. The safety of children is always an important issue for both caregivers and healthcare providers. According to data from the World Health Organization, the adverse events resulting from unsafe medical practices is one of the top ten causes of death and disability in the world [45].

Caregivers reported unexpected changes in their children’s sleep, food intake, and defecation, along with the improvement of respiratory symptoms, which aligns with previous qualitative studies on pediatric massage. In the ADHD study in Hongkong, improved sleep quality and increased appetite were the most notable changes described by parents [43]. Similar improvements in sleep have been reported in children with cancer [46], hematopoietic cell transplantation [47], and disabilities [48] after pediatric massage. The improvement of bowel movements was also found among children with cerebral palsy after massage [49]. Clinically, these practices stimulate vagal activity through baroreceptors and mechanoreceptors in the skin, leading to a holistic effect [50]. These perceived bodily changes suggest that when receiving PT, multiple embodied reactions occur, which are extra benefits for both children and caregivers. Although sleep improvements are reported independently from changes in coughing by some of the caregivers, it is important to be aware that in cases where improvements in sleep and cough were reported together, improved sleep may be the result of reduced coughing [51].

Regarding the changes in coughing, it is challenging to untangle the specific factor. As some mothers reflected in this study, there could be several factors, such as the improved sleep and food intake, the children’s natural growth or the PT. Previously, we described how reduced coughing can have a positive effect on sleep, and vice versa, improved sleep or a healthier diet may prevent children from new episodes of RRTIs [52]. We know that the incidence of respiratory tract infections decreases gradually with age [53]. A survey study in Italy demonstrated that children between the ages of 6 months and 3 years were more susceptible to acute respiratory infections [54]. However, for some certain pathogens, such as influenza A virus and influenza B virus, children older than 3 years were at a higher risk of being infected compared to children younger than 3 years [55, 56]. In this study, the children involved ranged in age from 3 to 6 years old. We feel uncertain to solely attribute the improvement in cough to the natural process of aging. To identify the effect size of PT in preventing RRTIs among children, randomized controlled trials with large sample sizes are needed.

In addition to the physical changes, caregivers reported improved attachment between children and adults as a result of PT. This finding aligns with previous qualitative studies on parents’ experiences with pediatric massage or touching therapy in other pediatric health-related situations. In children who had received hematopoietic cell transplantation, parents reported that they felt a sense of intimacy and connection with their child when they performed massage [47]. Infant massage serves as a conscious means by which mothers can bond with their babies [57]. In another infant massage study, the mothers recognized the connection they made with their infant through massage as the attachment relationship [58]. Furthermore, parents of children with autism believed that touch therapy established a communication channel, facilitating interaction between them and their children [59]. All of this researches indicates that the embodied manual therapies, such as PT, have in the benefit of nurturing the attachment between children and caregivers.

Caregivers also observed the emotional link between their children and the practitioner. A qualitative study regarding the psychotherapeutic relationship in massage therapy indicated that a key function of the therapist role is to facilitate pleasure, which is constituted by safety, comfort and communication [60]. These elements also fit into the context of this study, where PT practitioners provided gentle, soft manipulations and a comfortable environment. The relationship between children and PT practitioners is built on both tactile and verbal communication.

In this study, some caregivers showed an interest in learning PT and integrating PT into their family life. It is accessible for parents to learn PT in China. They may learn PT from the PT providers. During the PT session, they are allowed to take videos of the manipulations for later review and they are free to ask any questions regarding PT. Caregivers may also learn PT through the Internet. However, when they use online resources, it is important that the caregivers to make sure that they are learning from professional PT providers. When applying PT to their children at home, the most important thing is the safety. Hand hygiene is on the top of the list. Rings and bracelets should be taken off before PT to avoid scratching the child’s skin. In addition, massage oil is recommended to protect the skin and also to help the caregivers implement the manipulations smoothly. In addition to the concerns of safety, it is also important to consider the children’s feeling. When the children is full, hungry or crying, it is better not to administer PT.

### Strengths and limitations

One strength of this study was that caregivers’ experiences were freely discussed in the interviews, which provided in-depth data to analyze. Furthermore, the sample included both children who had received PT before the current trial and those with no previous experience with PT. Due to COVID-19, all of the interviews were conducted online, which might be a limitation. Nevertheless, all caregivers were acquainted with the digital tools and expressed no adverse feedback regarding the format. Another limitation is that the study does not include the experiences of fathers. However, in China, mothers spend more time with their children and tend to provide more detailed information about them. The sampling of the caregivers was limited to participants of a clinical trial. It would have been better to also enroll caregivers whose children receive PT within a regular clinic context, which would better reflect real life experiences. Unfortunately, this was not possible due to COVID-19. We cannot exclude recall bias in the later interviews. We tried to minimize this bias when we designed the interview guide by starting with questions that would help participants remember their disease stories.

## Conclusions

Generally, caregivers had a positive experience with PT. Their embodied observations of PT indicate that they consider PT to be safe for their children and may potentially have some positive physical effects beyond those related to the symptoms of RRTIs, such as improved appetite, sleep and defecation. Additionally, PT is seen to serve as a means of interaction between children and adults, fostering embodied care on both a physical and emotional level. An implication of this study for future practice is that beyond its potentially preventive effect on RRTIs, PT could be a supportive tool, to be used at home by parents of children with recurrent or chronic disease [43].

## Electronic supplementary material

Below is the link to the electronic supplementary material.

Supplementary Material 1

## Data Availability

Data are accessible upon reasonable request, and anonymized transcripts can be obtained from the first author (LY).

## Abbreviations

PT: Pediatric Tuina (massage)
RRTIs: Recurrent respiratory tract infections
TCIH: Traditional, complementary, integrative healthcare
TCM: Traditional chinese medicine
COREQ: Consolidated Criteria for Reporting Qualitative Research
