# Supplementary material for: “She mimicked the manipulations on my hand”: fostering embodied care among children with recurrent acute respiratory tract infections in Southern China
Source: BMC Complement Med Ther. 2024 Oct 7;24:359. doi: 10.1186/s12906-024-04660-6 (PMC11457486; doi:10.1186/s12906-024-04660-6)
Supplement: Supplementary file 1 — Supplementary Material 1 [file 12906_2024_4660_MOESM1_ESM.docx]

**Supplementary table 1. Interview Guide**

| **Topics** | **Interview questions** |
| --- | --- |
| Warm-up questions | How old is your child?  Boy or girl?  Usually, who takes care of him/her?  How many people are there living together in your family?  What are the family members?  What’s your job? |
| The disease story of the child | How was your child health condition, before receiving the PT therapy?  Can you please tell me the story of your child RRTIs? For instance, when did your child start to have this problem? What did you do? What you thought was the cause? What were you worried about?（worries within one episodes and worries regarding the recurrent infections).  ( What was the occurrence frequency? What was the main symptom? etc.) |
| The health care seeking experience | When your child was ill and how did you decide it was time to seek health care? For instance, Under what circumstances would you feel unable to continue observing the child at home?  Where did you usually go to seek health care for your child? How did you decide where to go?  How do you feel when you take your child to see a health care provider?  Can you describe me one visiting experience?  What are your opinions on biomedicine and Chinese medicine?  What makes you decide to take your child to see a biomedicine provider or a Chinese medicine provider. |
| Impression of Pediatric *Tuina*(PT) | Have you heard of PT before this trial?  What’s your first impression of PT? |
| Experiences with PT | Can you describe me a PT experience? For instance how was the treatment? The setting, the staff and the use of hands or the use of medicines? How do they prepare the child?  How did you feel when your child received the PT?  In your perspective how did your child feel during the PT process?  After completing the try, has your understanding of PT changed in any way?  What prompts you to choose PT? |
| Outcomes after PT | How is your child health condition now, after the one-month PT therapy?  Compared with the condition before, what are the changes in terms of your child’s eating, sleeping and defecation condition?  In addition to the above aspects, what are the other changes on your child |
| Reasons of participating in the trial | Why you decided to be part of the research trial?  What were your considerations in joining / not joining?  Who discussed and decide? Did you ask your families or friends?  Have you participated in other research trial? |
| Ending | We have been talking about the story of your child’s RRTIs, the reasons why you decided to participate in the research trial, your understandng , experience and feeling about PT. I learned a lot from you, thank you. Before we end this conversation today, I would like to ask you do you have any other things that I haven’t asked about and you would like to share?  Is it ok that I talked to you in the future, if I find some information is missing? |
